# Supplementary material for: Isolation and long-term expansion of murine epidermal stem-like cells
Source: PLoS One. 2021 Jul 16;16(7):e0254731. doi: 10.1371/journal.pone.0254731 (PMC8284819; doi:10.1371/journal.pone.0254731)
Supplement: S1 File — (PDF) [file pone.0254731.s001.pdf]

Supplemental Materials and Methods

Global gene expression. The quality of total RNA was QC analyzed by Bioanalyzer (Agilent, Santa Clara, CA). To isolate poly A RNA for library preparation, NEBNext Poly(A) mRNA Magnetic Isolation Module (New England BioLabs, Ipswich, MA) was used with 1 µg good quality total RNA as input. The poly A RNA was enriched using SMARTer Apollo automated NGS library prep system (Takara Bio USA, Mountain View, CA). NEBNext Ultra II Directional RNA Library Prep kit (New England BioLabs) was used for library preparation under PCR cycle number of 8. After library QC and quantification via real-time qPCR (NEBNext Library Quant Kit, New England BioLabs), individually indexed libraries were proportionally pooled and sequenced using NextSeq 550 sequencer (Illumina, San Diego, CA) under the sequencing setting of single read 1x85 bp.

For sequencing reads generation, 23.4 +/- 1.6 (mean +/- SE) million pass filter reads per sample were generated. For each sample, >97% reads aligned to MM10 reference genome, >99% stranded, and ~85% reads aligned to coding and untranslated region (UTR), which indicated good data quality.

Data analyses. Fastq files for downstream data analysis were automatically generated via Illumina BaseSpace Sequence Hub. To identify differentially expressed genes, standard bioinformatic analysis was performed via BaseSpace app RNA-Seq Alignment v2.0.2 followed by RNA-Seq Differential Expression app version 1.0.1. For read alignment, reference genome Mus. Musculus/MM10 (RefSeq) was used. The alignment was performed under 1st strand setting, and the analysis used STAR for alignment and Salmon for quantification. STAR produced a BAM and transcriptome.BAM file and Salmon uses the transcriptome. BAM file and assigns Transcripts Per

Million (TPM) to genes and transcripts. The alignment result was then seamlessly used as input in RNA-Seq Differential Expression app, which performed differential expression analysis of reference genes with DESeq2. Significant genes were selected based on adjusted p-value <0.01.
