# Supplementary material for: Isolation and long-term expansion of murine epidermal stem-like cells
Source: PLoS One. 2021 Jul 16;16(7):e0254731. doi: 10.1371/journal.pone.0254731 (PMC8284819; doi:10.1371/journal.pone.0254731)
Supplement: S2 File — (DOCX) [file pone.0254731.s002.docx]

**S1 Table. Antibodies**

| **Name** | **Host species** | **Antibody type** | **Cata No** | **Company** |
| --- | --- | --- | --- | --- |
| Keratin 14 (Krt14) | Mouse | monoclonal | MA5-11599 | Thermo Fisher |
| Keratin 1 (Krt1) | Rabbit | Polyclonal | 905602 | BioLegend |
| Involucrin (Ivl) | Rabbit | Polyclonal | 924401 | BioLegend |
| Loricrin (Lor) | Rabbit | Polyclonal | 905101 | BioLegend |
| Filaggrin (Flg) | Rabbit | Polyclonal | 905801 | BioLegend |
| Zonula occludens-1 (ZO-1) | Rabbit | Polyclonal | 61-7300 | Themo Fisher |
| Ki67 | Rabbit | Polyclonal | PA5-19462 | Themo Fisher |
| E-Cadherin (E-Cad) | Mouse | Monoclonal | 610181 | BD |
| pIkBα | Mouse | Monoclonal | 551818 | BD |
| Pai-1 | Mouse | Monoclonal | 528717 | Calbiochem |
| PCNA | Mouse | Monoclonal | 555566 | BD |
| β-Actin | Mouse | Monoclonal | A5316 | Sigma |
| Mouse IgG HRP | Goat | Polyclonal | 31430 | Thermo Fisher |
| Rabbit IgG HRP | Goat | Polyclonal | 31460 | Thermo Fisher |
| Rabbit IgG Alexa Fluor 488 | Goat | Polyclonal | A11034 | Thermo Fisher |
| Mouse IgG Alexa Fluor 568 | Goat | Polyclonal | A11004 | Thermo Fisher |

**S2 Table. Cell culture reagents**

| **Name** | **Cata No** | **Company** |
| --- | --- | --- |
| KSFM-Ca^2+^ | 37010-022 | Gibco |
| KSFM+Ca^2+^ | 17005-042 | Gibco |
| DKSFM | 10744019 | Gibco |
| DMEM | 10-017-CV | Corning |
| Fetal bovine serum | S11150 | Atlanta Biological |
| Knockout™ Serum | 1082028 | Gibco |
| L-glutamine | 25-005-Cl | Corning |
| Non-essential amino acid | 11140-050 | Gibco |
| Sodium pyruvate | 11360-070 | Gibco |
| TrypLE | 12604-021 | Gibco |
| 0.25% Trypsin | 15050-057 | Gibco |
| Gelatin | G-2500 | Sigma |
| Basement Membrane Extract | 3445-005-01 | Cultrex |

**S3 Table. Primers sequences in the qPCR**

| **Gene** | **Forward (5'-3')** | **Reversre (5'-3')** |
| --- | --- | --- |
| *Gapdh* | AACGACCCCTTCATTGACC | TGAAGACACCAGTAGACTCC |
| *Krt 14* | GAGCGGCAAGAGTGAGATTT | CTTTGGTCTCCTCCAGGTTATTC |
| *Krt1* | TTCATCGACAAGGTGCGCTTCCTA | TGGTCACGAACTCATTCTCTGCGT |
| *Krt 10* | CTCACCCTGACAACTGACAAT | CCCTGAAGTCGAGGAGCT |
| *Krt 8* | CTGGTGGAGGACTTCAAGAATAA | ATGCTTCGTCCACATCCTTC |
| *Krt 15* | ACATGCTGCTGGACATCAA | GGGATACTTCTCTGACACCAATAC |
| *Krt 18* | TGCAGCTGGAGACAGAAATC | ATCCACTTCCACAGTCAATCC |
| *Lor* | AACGGAGACAACAGAGCTGGAAGA | ACCTTGAGCGACTCAATGGCTTCT |
| *Cd34* | AAGGCTGGGTGAAGACCCTTA | TGAATGGCCGTTTCTGGAAGT |
| *Sca-1* | AGGAGGCAGCAGTTATTGTGG | CGTTGACCTTAGTACCCAGGA |
| *Pai1* | CTCCAATTACTGGGTGAGTC | GGTGGAGACATAACAGATGC |
| *Ccl2* | TCACCTGCTGCTACTCATTCACCA | AAAGGTGCTGAAGACCTTAGGGCA |
| *Pcna* | TTTGAGGCACGCCTGATC C | GGAGACGTGAGACGAGTCCAT |
| *Ki67* | ATCATTGACCGCTCCTTTAGGT | GCTCGCCTTGATGGTTCCT |
| *Cdh1* | CAGGTCTCCTCATGGCTTTGC | CTTCCGAAAAGAAGGCTGTCC |
| *p16^INK4a^* | AATCTCCGCGAGGAAAGC | GTCTGCAGCGGACTCCAT |
| *Tel* | CGGTTTGTTTGGGTTTGGGTTTGGGTT TGGGTTTGGGTT | GGCTTGCCTTACCCTTACCCTTACCCT TACCCTTACCCT |

**S4 Table. Inflammation gene expression levels in RNA-Seq.**

| **Gene** | **log2FoldChange** | **KSFM-Ca^2+^** | **DKSFM** | **padj** |
| --- | --- | --- | --- | --- |
| *Il1a* | -8.050163001 | 2553.537657 | 9.633891113 | 2.48E-179 |
| *Ccl2* | -7.596804638 | 2531.671298 | 13.07799752 | 3.90E-212 |
| *Cxcl1* | -7.391804178 | 20573.14114 | 122.5027466 | 0 |
| *Cxcl5* | -7.067457776 | 7092.924311 | 52.88208159 | 0 |
| *Bst1* | -5.85374095 | 4188.974442 | 72.43627318 | 0 |
| *Cxcl3* | -5.013849771 | 2735.902851 | 84.68012633 | 7.75E-143 |
| *Ppbp* | -4.899449332 | 4316.569779 | 144.6297231 | 6.72E-110 |
| *C3* | -3.354955269 | 3468.795531 | 339.0287519 | 0 |
| *Serpine1* | -3.262878595 | 2505.200555 | 260.9865761 | 1.99E-120 |
| *Gpr137b* | -2.80397511 | 1962.632808 | 281.0337246 | 3.25E-288 |
| *Ikbke* | -2.697954858 | 2665.567612 | 410.7942573 | 0 |
| *Dapk2* | -2.439026295 | 1948.916649 | 359.3959598 | 2.23E-244 |
| *Tfrc* | -2.368578444 | 6828.243983 | 1322.196101 | 0 |
| *Ier3* | -2.32968672 | 4885.741846 | 971.9074979 | 0 |
| *Nfkbia* | -2.291033711 | 4302.22496 | 879.0695717 | 0 |
| *Ercc1* | -2.12607079 | 1812.573694 | 415.2260501 | 2.27E-200 |
| *Plau* | -2.080155153 | 4532.39846 | 1071.862277 | 0 |
| *Itgb6* | -1.922654246 | 2350.481541 | 619.9836631 | 1.37E-180 |
| *Xrcc5* | -1.860027943 | 2570.65936 | 708.1419591 | 7.98E-216 |
| *Uaca* | -1.831456112 | 1615.127215 | 453.8198726 | 1.80E-154 |
| *Tmem176b* | -1.681645364 | 4086.309974 | 1273.812807 | 6.91E-273 |
| *Pola1* | -1.678530794 | 2315.032744 | 723.2176566 | 9.11E-173 |
| *B2m* | -1.674161548 | 2824.670286 | 885.1052518 | 9.22E-169 |
| *Pbk* | -1.66706973 | 3064.111803 | 964.8651363 | 3.23E-231 |
| *Phldb2* | -1.634083714 | 4513.573426 | 1454.16035 | 4.71E-284 |
| *Epha2* | -1.623213893 | 3215.914635 | 1043.92294 | 1.21E-208 |
| *Slc12a2* | -1.506022705 | 5530.010705 | 1947.00902 | 7.62E-278 |

Log2FoldChange represented Log2 Fold (DKSFM vs KSFM-Ca^2+^). Average TPM counts to show the gene expression levels. Adjusted p-value (padj)<0.01 was used to cut off.
