# Supplementary material for: Isolation and long-term expansion of murine epidermal stem-like cells
Source: PLoS One. 2021 Jul 16;16(7):e0254731. doi: 10.1371/journal.pone.0254731 (PMC8284819; doi:10.1371/journal.pone.0254731)
Supplement: S3 File — (PDF) [file pone.0254731.s003.pdf]

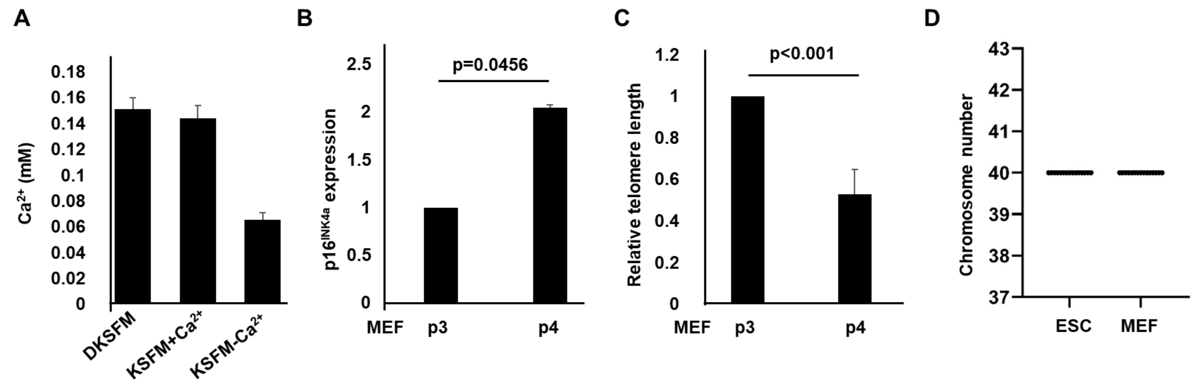

S1 Fig. Growth media and control cell characterizations. (A) The calcium levels in DKSFM, KSFM+Ca^{2+} and KSFM-Ca^{2+} media were measured using the Calcium Concentration Kit and results represent average of 3 biological samples of each medium. (B) *p16*^{INK4a} mRNA expression in MEF passage 3 and 4 (p3 and p4). Set early passage p3 as 1. (C) Relative telomere length between MEF passage 3 and 4 (p3 and p4). (D) Normal chromosome number (40) in mESC and MEF.

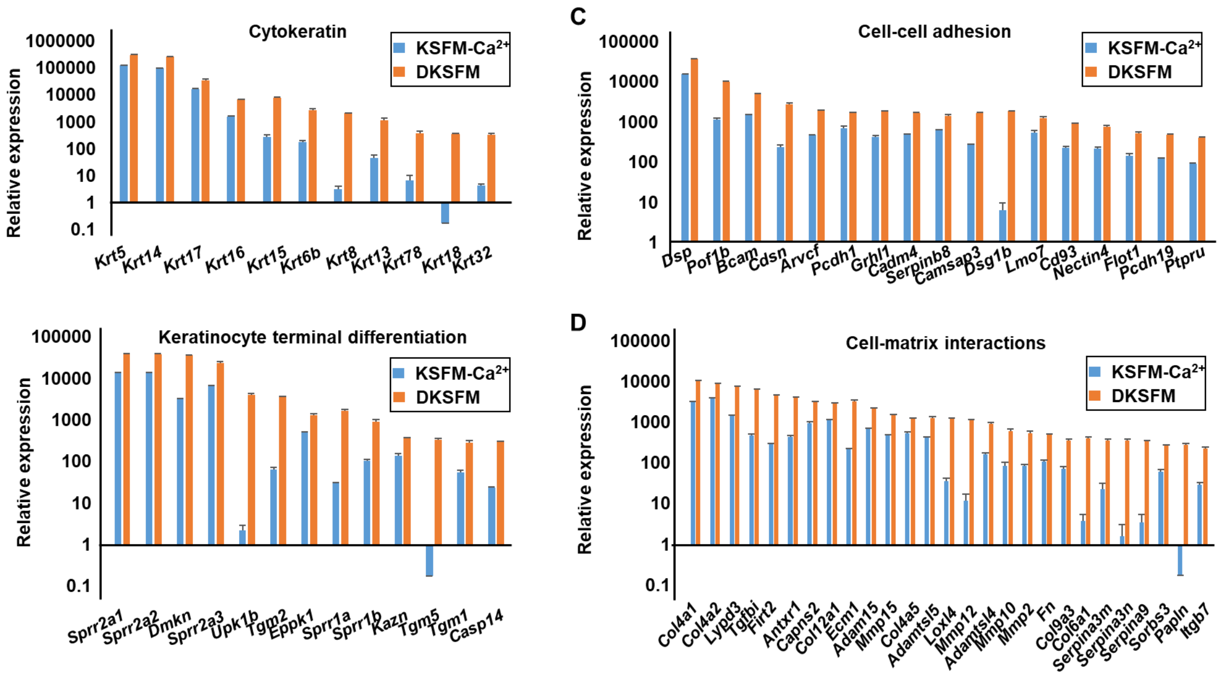

S2 Fig. Functional groups of genes up-regulated in the DKSFM cells. RNA-Seq data were normalized counts using DESeq2. Transcripts per kilobase million (TPM) were used to evaluated the expression levels of genes in the groups of **(A)** cytokeratin markers, (B) keratinocyte terminal differentiation, (C) cell-cell adherence, and (D) cell-matrix interactions.

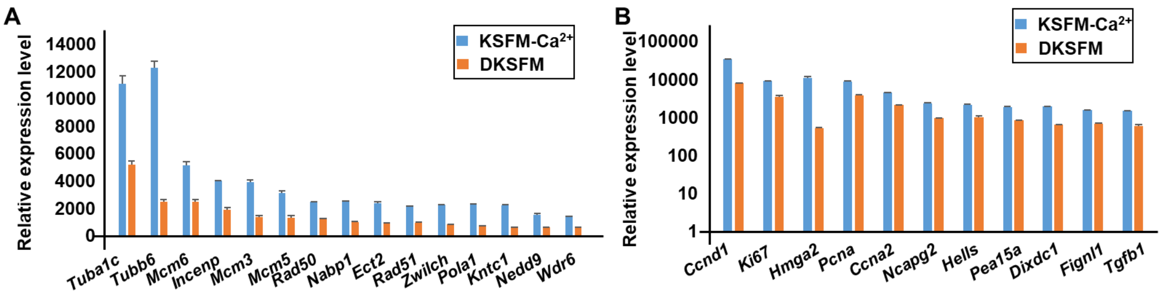

S3 Fig. Functional groups of genes up-regulated in the KSFM-Ca^{2+} cells. RNA-Seq data were normalized counts using DESeq2. Transcripts per kilobase million (TPM) were used to evaluated the expression levels of genes in the groups of (A) immune response and (B) cell proliferation.

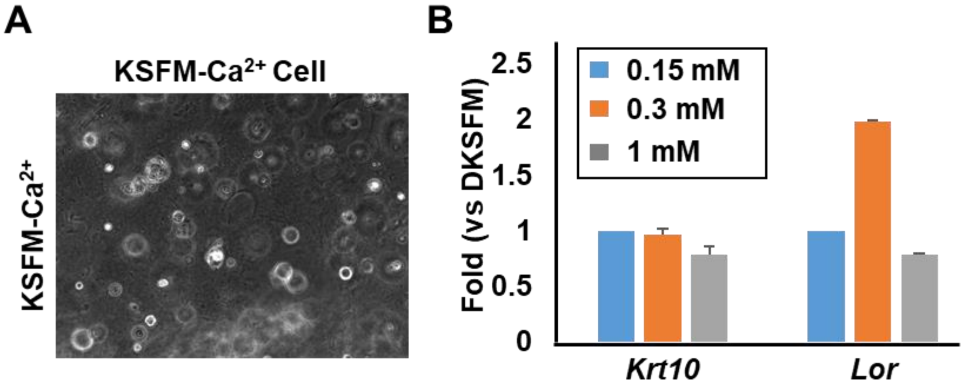

S4 Fig. Terminal differentiation potential of the cell lines. (A) KSFM-Ca^{2+} cells grown in BME up to 14 days were unable to form organoids in the KSFM-Ca^{2+} medium. (B) When cultured in 2D conditions, the DKSFM (0.15 mM Ca^{2+}) cells did not have increased expression of *Krt10* and *Lor* in media with increased extracellular calcium, suggesting that high Ca^{2+} alone was insufficient to induce terminal differentiation.
