# Supplementary material for: Isolation and long-term expansion of murine epidermal stem-like cells
Source: PLoS One. 2021 Jul 16;16(7):e0254731. doi: 10.1371/journal.pone.0254731 (PMC8284819; doi:10.1371/journal.pone.0254731)

S1 Raw images. Original blot of Fig. 6C. Cells were lysed in RIPA buffer; 50 µg lysates were subjected to SDS–PAGE. Proteins on the SDS-PAGE gels were transferred onto nitrocellulose membranes and probed with antibodies. Anti-PAI-1 (A) and anti-pIκBα (B) antibodies were diluted 1:1000. Anti-β-Actin (C) diluted at 1:5000 was used as loading control. Anti-mouse-HRP and anti-rabbit-HRP antibodies were diluted in 1:2000. The membranes were incubated with West Pico PLUS Chemiluminescent Substrate (Thermo Scientific) and the signal was captured by UVP BioImaging system. * was represented target protein bands.

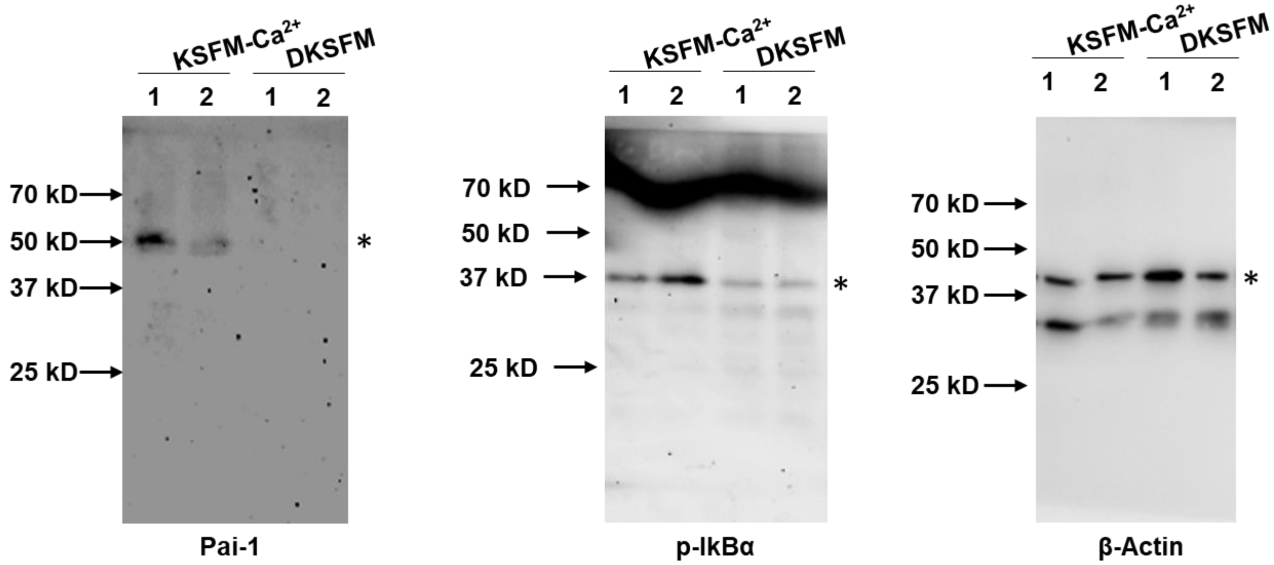

Supplement: S1 Raw images — (PDF) [file pone.0254731.s004.pdf]
