# Supplementary material for: Isolation and long-term expansion of murine epidermal stem-like cells
Source: PLoS One. 2021 Jul 16;16(7):e0254731. doi: 10.1371/journal.pone.0254731 (PMC8284819; doi:10.1371/journal.pone.0254731)
Supplement: Attachment — Submitted filename: cover letter-plos one resubmission.pdf [file pone.0254731.s005.pdf]

UNIVERSITY OF Cincinnati

Department of Environmental and Public Health Sciences
University of Cincinnati
PO Box 670056
Cincinnati, OH 45267-0056

Kettering
3223 Eden Avenue
Cincinnati, OH 45267-0056
(513) 558-1284

June 21, 2021

Dear Dr. Lako,

Thank you for giving us the opportunity to resubmit the manuscript, entitled "Isolation and long-term expansion of murine epidermal stem-like cells" by Wang et. al. (PONE-D-21-10675). We also thank the reviewer for the critiques, based on which we have revised the paper with new experimental data. Herein, we provide point-by-point responses to the reviewer's comments and outline changes made in adherence to Journal's requirements:

1. Their data imply a degree of stem cell like characteristics in the cell population they generate (eg CD34, CD200, expression) but they have not indicated why such stem cells have this proliferative capacity. It would be useful to measure telomere lengths, expression of mTert and activity of the murine telomerase holoenzyme complex in addition to markers of cell senescence such as expression of p16 INK4a.

   Authors' responses: The keratinocyte lines reported here displayed some stem cell characteristics, including unlimited self-renew and prolonged *in vitro* culture capabilities, in addition to the expression of stem cell genes. Our new experiments, as suggested by the reviewer, showed that neither $p16^{INK4a}$ expression nor telomere length were significantly altered in cells at low and high passages. suggesting long-term stability of these cells.

2. They do not provide analyses of the karyotype at early and late passage numbers.

   Authors' responses: This was an excellent point we had omitted previously. Karyotyping showed that even the primary mouse keratinocytes (P0) had significantly increased chromosomes consistent with what was reported before (Hammiller, 2015). We detected, accordingly, tetraploid or hyperdiploid chromosomes in our keratinocyte lines in both the early and late passages. These observations suggest that the chromosomes, originally unstable in primary keratinocytes, became relatively stable in the cell lines.

3. Another drawback of the data provided is that there seems to be no attempt to correlate transcriptomic identity of the cell population to passage number. Given that doubling times decrease, it may be assumed that the transcriptome of the bulk population does change so taking a "snapshot" of gene expression does not provide an accurate description of a potentially changeable phenotypic environment.

   Authors' responses: With the additional data on telomere, p16 INK4a expression and chromosomal numbers, and the fact that the doubling time became stable in cells at "high

(>30) passages”, we reason that the high passaging cell lines maintain stable molecular signatures, and hence, gene expression.

4. The following changes were made in accordance to Journal requirements:
   (1) Revised methods and specified methods of euthanasia
   (2) Removed “data not shown”
   (3) Included original images for blots in Supporting Information files.

Please feel free to contact me if you have any questions.

I look forward to hearing from you.

Sincerely yours,

Ying Xia, Ph.D.
Professor
Department of Environmental and Public Health Sciences
University of Cincinnati

Phone: 513-558-0371
Email: ying.xia@uc.edu
